# Detection of Single Umbilical Artery in the First Trimester Ultrasound: Its Value as a Marker of Fetal Malformation

**DOI:** 10.1155/2014/548729

**Published:** 2014-07-03

**Authors:** Cristina Martínez-Payo, Elena Cabezas, Yolanda Nieto, Miguel Ruiz de Azúa, Fátima García-Benasach, Enrique Iglesias

**Affiliations:** Department of Obstetrics and Gynecology, Prenatal Diagnosis Unit, University Hospital Puerta de Hierro-Majadahonda, C/Manuel de Falla N°1, Majadahonda, 28222 Madrid, Spain

## Abstract

*Introduction*. The value of a single umbilical artery (SUA) in first trimester ultrasound is not well established. The aim of our study was to determinate the relevance of diagnosis of single umbilical artery in first trimester ultrasound as an early marker suggesting the presence of malformations or associated chromosomopathies. *Material and Methods*. Retrospective study of clinical cases of SUA diagnosed at the University Hospital Puerta de Hierro in Madrid (Spain) during the first trimester ultrasound between September 2008 and September 2012. *Results*. Prevalence of SUA was 1.1% in single pregnancies and 3.3% in twin pregnancies. Sensitivity, specificity, false positive rate, and false negative rate for the finding in the first trimester were 84.2, 99.8, 0.2, and 15.7%, respectively. 17.6% of cases had associated malformations. With an ultrasound in the 16th week most of the cases with significant fetal malformation were diagnosed. *Discussion*. SUA is a useful marker in the first trimester for fetal malformation pathology, as it will allow detecting a large number of cases with malformations before 20 weeks of gestation.

## 1. Introduction

The umbilical cord contains two arteries and one vein. The absence of one of the arteries is called single umbilical artery (SUA). It is one of the most common sonographic findings during pregnancy with an incidence range that goes from 0.5 to 6 percent in single pregnancies [1–3]. This incidence increases three or four times in twins pregnancies [4, 5].

The association of SUA with fetal anomalies, mainly genitourinary and cardiac, with or without genetic alteration has already been studied. SUA is also associated with intrauterine growth restriction, preterm delivery, and poor obstetric outcomes. However, SUA as an isolated sonographic finding may be related to a normal neonatal outcome [6–8].

SUA in second and third trimesters has been repeatedly studied, but in the first trimester the search of the number of cord vessels, although it is recommended, is not normally part of the routine [9].

The aim of our study is to determinate the reliability of the diagnosis of SUA in the first trimester ultrasound and its relevance as a marker of malformations or genetic disorders, as well as knowing if isolated SUA in the first trimester allows reassuring parents or if otherwise it keeps the uncertainty about the development of pregnancy.

## 2. Material and Methods

We performed a retrospective study of all cases of SUA diagnosed during the 12th week ultrasound, between September 2008 and September 2012, at the Prenatal Diagnosis Unit at the University Hospital Puerta de Hierro in Madrid. The ultrasound scans were carried out by a Voluson 730 Expert system, GE Medical Systems, Zipf, Austria. First trimester sonography was performed by senior obstetrician specialists in Prenatal Diagnosis. In ultrasonographic assessment of 12 weeks, we seek the systemic assessment of placentation, the number of vessels in the umbilical cord, the cord insertion into the placenta, the subjective evaluation of amniotic fluid, the fetal biometry with a CRL, the measurement of the nuchal translucency, the assessment of the nasal bone, the ductus venosus, and the tricuspid regurgitation index. All this information allows us to estimate the risk of chromosomal abnormalities in our patients by combining the first trimester screening and a full fetal anatomical study (like the ultrasound made in the 20th week, although considering the limitations of gestational age). First trimester ultrasound was performed by abdominal and vaginal routes, in order to visualize all structures described above. Approximately, 95% of first trimester scans required both ways.

**Figure 1:**
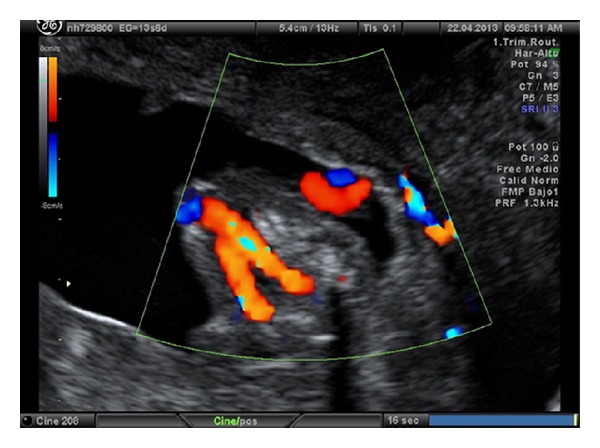
Image at the level of the fetal pelvis. We can see both umbilical arteries are displayed surrounding the fetal bladder and going to the abdominal wall.

**Figure 2:**
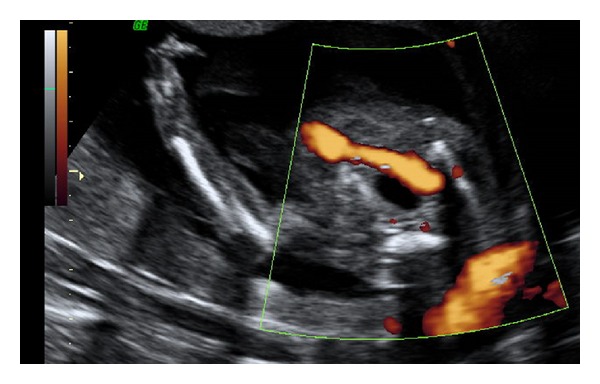
Image at the level of the fetal pelvis. We can see a single umbilical artery surrounding the fetal bladder.

The technique used to evaluate the number of cord vessels in the first trimester ultrasound was the identification, using Doppler color, of the number of arteries around both sides of the bladder wall, in a cross section at the fetal pelvis level before they join in the anterior abdominal wall to become part of the umbilical cord, along with the umbilical vein. If it was necessary we used the bidirectional power Doppler. If one of these arteries was not found, a SUA diagnosis was established (Figures 1 and 2). In subsequent scans, the number of umbilical cord vessels was assessed using the same technique described above or quantifying the number of umbilical arteries in a cross section of the cord into a free loop [4]. Invasive diagnostic techniques were recommended only if there was a significant morphological alteration accompanying this finding. On the other hand, if it was considered an isolated finding, the patient was placed to a complete pre-20th week ultrasound that was usually done in the 16th week.

For the study, only low and high risk pregnancies were evaluated, in which at least an ultrasound examination was performed in the 12th and 20th weeks of gestation. We identified from our database patients that in the 12th week ultrasound were diagnosed with SUA, and in those cases, we establish whether any morphological alteration or marker of abnormal chromosomes was present, and if a genetic study had been conducted we also considered its results. To evaluate the false positive and negative diagnosis of SUA in the first trimester, this scan was compared with the one made in the 20th week. The followup of the cases was done by consulting obstetric history, and in the cases where birth took place, pediatric assessment of the newborn during the days of postpartum was also consulted. In cases where the followup in our hospital was not complete, we contacted the patients by telephone, being excluded from the study if this was not possible.

## 3. Results

A total of 10008 fetuses were analyzed between September 2008 and September 2012 and were studied by ultrasound, at least in the 12th and 20th weeks of gestation. Among those, 108 fetuses were diagnosed with SUA in the first trimester ultrasound. This means an ultrasound prevalence of SUA of 1.1% in our population. 17 cases were considered first trimester's false positives, as in the 20th week ultrasound two arteries were visualized. Also, 17 fetuses with apparently normal umbilical cord in the 12th week were afterwards diagnosed with SUA. 91 cases were truly diagnosed with SUA in the 12th week ultrasound; 8 developed into miscarriage or legal abortion, before an ultrasound confirmation was performed (Table 1, cases 9–16). The sensitivity and specificity of first trimester ultrasound for SUA were 84.2% and 99.8%. The positive predictive value was 84.2% and the negative predictive value was 99.8% with a false negative rate of 15.8% and false positive rate of 0.2%. Seven fetuses were from a twin pregnancy, in which one of the two twins was affected. During the studied period, a total number of 213 multiple pregnancies were described; then, the prevalence of SUA among multiple pregnancies was 3.3%.

16 fetuses had concomitant malformation associated with the SUA diagnosis, in the 12th week ultrasound, which represents a 17.6% of the cases (Table 1, cases 1–16). In cases from 17 to 22 in Table 1, subsequent ultrasound, performed between weeks 14th and 20th, showed relevant findings, which in 4 cases led to the termination of the pregnancy. One other case was a diamniotic dichorionic twin pregnancy, where we counseled to wait for spontaneous evolution due to the adverse prognosis and high fatality rate of intrauterus trisomy 18, expecting the correct evolution of the nonaffected fetus. Of the 17 false negative patients none had any other ultrasound findings in the following studies.

18 fetuses showed other abnormalities in the ultrasound. Those had a chromosomal disorder study made by analyzing karyotype. Five of these patients had an altered karyotype, and the trisomy 18 was the most frequent one.

## 4. Discussion

The association of SUA with intrauterine growth restriction [6], preterm delivery, fetal poor obstetric outcomes, and congenital anomalies has been described. The most frequently associated congenital anomalies are genitourinary and cardiac, but the SUA has also been associated with other anomalies such as gastrointestinal, central nervous system, and other less common ones as diaphragmatic hernia, fetal hydrops, musculoskeletal anomalies, exstrophy of cloaca sequence, sirenomelia sequence, or VATER syndrome [1, 3, 10].

The association with chromosomal defects occurs in approximately 10% of fetuses with SUA. The most common is the trisomy 18 [11], although trisomies 13 and 21 are also often found. Trisomy 21 does not appear to be associated with this anomaly, but it is found to be associated with this condition in most cases, due to the fact that it is the most frequent [2, 5, 12, 13]. In most of the cases there are other major defects, so that the finding of a single umbilical artery does not justify an amniocentesis, unless there are other associated ultrasound abnormalities [2]. SUA is more common in twin pregnancies, velamentous insertion, extreme maternal age, smoking, diabetic or hypertensive mothers, and seizure disorders [14, 15].

The number of cord vessels is evaluated since the 20th week ultrasound. The importance of searching for the number of cord vessels in the 12th week ultrasound is unclear and, in fact, does not belong to the usual clinical practice [9]. It seems that the display of the SUA in the first trimester may be more difficult than in the second trimester. The published values for sensitivity, specificity, and predictive values, both positive and negative, for the diagnosis are, respectively, 57.1%, 98.9%, 50.0%, and 99.2% in the first trimester and 86.6%, 99.9%, 92.9%, and 99.7% for the second trimester [16]. However, we found a rate of sensitivity, specificity, and positive and negative predictive values, similar to those reported for the second trimester. For this we used the vaginal route when it was necessary, and we also used the help of a bidirectional power Doppler. In any case, the prevalence of SUA during pregnancy is 1.1% in our unselected population, which coincides with literature [4, 16] when the search of the number of cord vessels is performed in a 20th–22nd week ultrasound. The prevalence found in twin pregnancies was 3.3%, that is, three times more than in singleton pregnancies, and is consistent with the published data [4, 5].

Considering that the mechanisms proposed to explain the embryogenesis of SUA are primary agenesis of one of the umbilical arteries, persistence of the original single allantoic artery of the body stalk, and secondary atresia or atrophy of previously normal umbilical artery [17, 18] it is possible that the reason for some of the false negatives is precisely this posterior atrophy, as we have checked in some patients after reviewing the images obtained at the 12th week ultrasound and at the following ones. In the 20th week ultrasound false positives and false negatives in ultrasound can appear when the umbilical cord of newborns is checked [19]. Despite this, we have considered the 20th week ultrasound as the gold standard to calculate the false positives and negatives of the 12th week ultrasound, as these findings will determinate the decisions we will make until the birth. This may introduce a small error in the statistical study, but our first trimester results are similar to those reported for the second trimester; it seems that SUA can be diagnosed similarly in both groups.

According to published studies, SUA is isolated between 64–96% of pregnancies [16]. In our series we found isolated SUA in 74.7% of the cases studied. 17.6% of fetuses with SUA in the first trimester had a fetal malformation that was sonographically evident in the 12th week ultrasound, and 7.7% of fetuses showed associated malformations in subsequent ultrasound performed; some of which could not have been diagnosed in the first trimester due to their evolving nature. In our opinion, the pursuit of the number of umbilical arteries before the 20th week ultrasound can have a great value. Now, in the 12th gestational week and aided by the use of the vaginal probe, it is possible to visualize fetal structures in a similar way to those obtained in later stages of pregnancy ultrasound, allowing advancing the diagnosis and advising parents. Performing an exhaustive review of fetal anatomy in the first trimester ultrasound, the cardiovascular system especially is currently possible. However, we must remain cautious when we have a diagnosis of isolated SUA in the first trimester, as there are fetal abnormalities that cannot be displayed, often because anatomical development has not yet been completed or because of the technique's limitations. An ultrasound in the 16th week has a great value; in fact, in our experience, five fetuses were diagnosed of a relevant pathology at this gestational age. Of course subsequent ultrasound guidance should be maintained, because other abnormalities in the central nervous system, digestive, urinary, and even heart may not be diagnosed until later stages; furthermore in these pregnancies it is necessary to control the fetal growth [1, 20].

Another point to consider is the use of Doppler ultrasound in early pregnancy. In our experience, detection of the number of vessels is rapid after suitable training, which keeps the ALARA principle.

In conclusion we can say that the assessment of the number of cord vessels during the 12th week ultrasound is useful because SUA can be considered a marker of fetal malformations diagnosable at this gestational age. Moreover, an isolated SUA in 12th week requires the completion of an ultrasound around the 16th week.

## Figures and Tables

**Table 1:** Clinical cases with SUA accompanying other major ultrasound abnormalities.

Number	Diagnostic weeks SUA	Findings in the 12th week ultrasound	Subsequent findings (from the 14th week to the finish of pregnancy)	Karyotype	Outcome
**1**	12 + 2	Tricuspid regurgitation, agenesis of the ductus venosus, and intestinal dilatation	16th week: interventricular communication	Normal	Preterm newborn (33rd week) with tracheal agenesis, intestinal pathology, and heart disease. He died after birth. Autopsy: VACTERL association.

**2**	12 + 2	Agenesis of the ductus venosus	20th week: interventricular communication, choroid plexos cysts	Normal	Preterm newborn (31st week) with congenital heart disease, imperforate anus, and external auditory canal atresia.

**3**	11 + 4	Increased nuchal translucency. Situs ambiguous, atrioventricular (AV) canal defects. Right atrial isomerism			Miscarriage (15th week)

**4**	12 + 4	Hypoplastic right ventricle. Tricuspid atresia/dysplasia			Loss to follow

**5**	12 + 2	Hygroma with chromosomal markers	16th week: Overriding aorta	Normal	Abortion (TOP)

**6**	12 + 1	Encephalocele, hydrocephalus, intestinal and liver herniation		Normal	Termination of pregnancy

**7**	12 + 0	Facial malformation	14th week: bilateral cleft lip, persistent right superior vena cava	Normal	Miscarriage (15th week)

**8**	11 + 5	Bladder augmentation, hydronephrosis, ARSA, ductus venosus position anomalies, increased nuchal translucency, polydactyly		T13	Miscarriage (13th week)

**9**	12 + 2	Truncus arteriosus, interventricular communication		T18	Termination of pregnancy

**10**	12 + 3	Hypoplastic left ventricle, cystic hygroma, and agenesis of the ductus venosus.		Normal	Termination of pregnancy

**11**	11 + 6	Fetal hydrops, omphalocele		45X0	Termination of pregnancy

**12**	12 + 3	Ectopia cordis, omphalocele, and amniotic bands		Normal	Termination of pregnancy

**13**	13 + 0	Caudal agenesis		Normal	Miscarriage (14th week)

**14**	11 + 0	Bilateral aplasia of forearm bones			Termination of pregnancy

**15**	11 + 6	Tricuspid regurgitation, pericardial effusion, and umbilical hernia		T18	Termination of pregnancy

**16**	11 + 3	Cystic hygroma, hypoplastic upper limb, and trident hand		Normal	Termination of pregnancy

**17**	13 + 1		17th week: cleft lip, pathology of the aorta	Normal	Termination of pregnancy

**18**	12 + 6		16th week: interventricular communication, ARSA 20th week: partial agenesis of the corpus callosum	T18	Stillbirth (31st week)

**19**	13 + 1		18th week: choroid plexos cysts. 25th week: hyperechoic vermis and tortuous umbilical vein	Normal	Normal newborn

**20**	12 + 4		17th week: hydrocephalus, bilateral agenesis of forearm bones	Normal	Termination of pregnancy

**21**	12 + 2		14th week: malformations of the upper limbs and hyperdense bowel		Termination of pregnancy

**22**	12 + 6		16th week: bilateral renal cysts	Normal	Termination of pregnancy
